# Active Monitoring of a Humeral Osteoblastoma in a 52-Year-Old Male: A Case Report

**DOI:** 10.14740/wjon871w

**Published:** 2015-04-12

**Authors:** Juliette Bouchet, Donia Lassoued, Nathalie Boussier, Jordan Birebent, Stephane Oustric, Marie-Eve Rouge-Bugat

**Affiliations:** a Toulouse Hospitals, France; b Paris Hospitals, France; c University Department for General Practice of Toulouse, France; d Department of Oncology at the Oncopole, Cancer University Institute, Toulouse, France

**Keywords:** Osteoblastoma, Osteoid osteoma, Humerus, Older than 30, Unusual case report

## Abstract

An osteoblastoma is an uncommon benign but painful tumor, typically found on the axial skeleton or on long bones in the case of young patients. Some cases of humeral osteoblastomas have been described in literature but not in men older than 30. We report the case of a painless bone tumor on the humerus of a 52-year-old patient. The CT scan shows a 30 mm hypodense lacunar formation, surrounded by thickened cortical bone resembling an osteoid osteoma. The anatomopathological and immunohistological analyses support the thesis of an osteoblastoma. A course of radiological monitoring without surgical resection was adopted. This unusual case introduces the possibility of carrying out a differential diagnosis with an osteosarcoma and raises the question of the treatment that should be adopted.

## Introduction

An osteoblastoma is an uncommon benign but painful tumor usually found on the axial skeleton or on long bones in the case of young patients. The presence of a painless osteoblastoma on the humerus is unusual in a patient over the age of 50.

## Case Report

**Figure 1:**
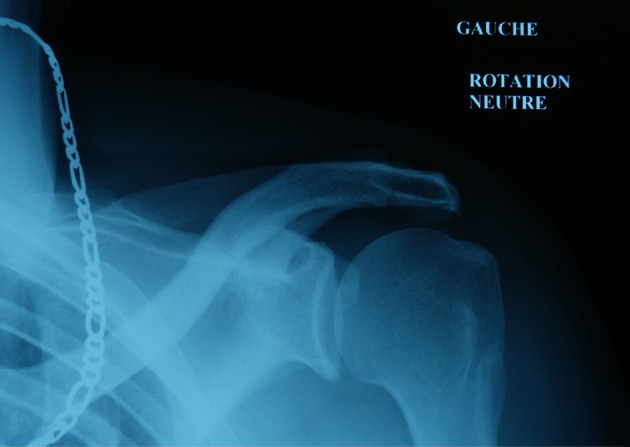
X-ray hypodense lesion in the cortical bone.

**Figure 2:**
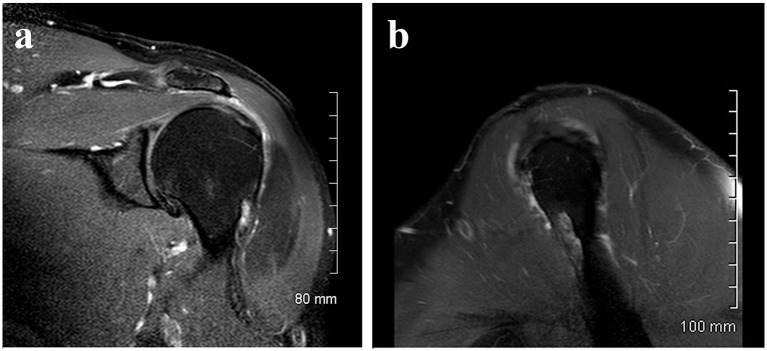
(a) MRI small geode under the cartilage of the humeral. (b) MRI small geode under the cartilage of the humeral.

**Figure 3:**
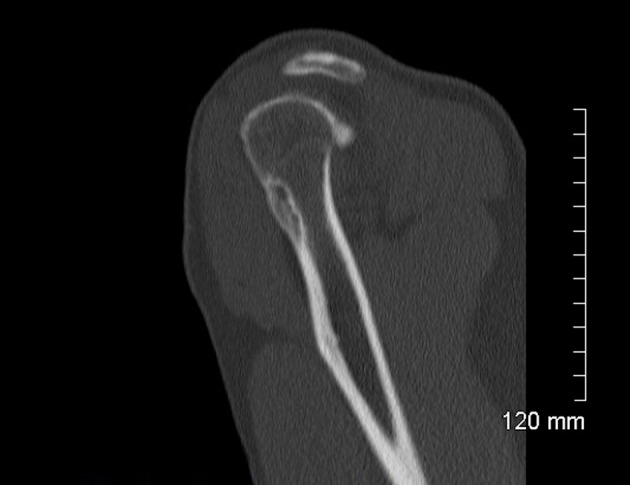
CT hypodense lacunar formation on the front cortical bone.

A 52-year-old male consulted for sudden swelling in the left deltoid region without his being aware of any recent traumatism. The clinical examination confirmed the painless swelling, which did not cause functional disability, suggesting a rotator cuff tear. An ultrasound did not show anything in particular. An X-ray found a hypodense lesion in the cortical bone on the upper third of the humerus (Fig. 1). The MRI confirmed a penetrating tear in the supraspinatus muscle and a lesion of the infraspinatus muscle. A “small geode” was found under the cartilage of the humeral head outside the bicipital groove (Fig. 2). The CT scan revealed a 30 mm hypodense lacunar formation on the front cortical bone of the upper humerus metaphysis, surrounded by thickened cortical bone resembling an osteoid osteoma (Fig. 3).

A CT-guided biopsy was carried out which identified a fibro-osseous lesion with osteoid bone surrounded by osteoclasts in the pathological anatomy. Between the osteoid bones, fairly dense fibroblastic tissue made up of spindle-shaped cells, without cytonuclear atypia, was identified. A large number of foamy macrophages and some giant multinucleate cells were also observed. There was an abundant blood supply. The immunohistochemical staining was positive with antigalectine 1, around the macrophagic cells with anti-CD68, and negative with anti-CDK4 and -MDM2. This was suggestive of an osteoblastoma without cytological evidence of malignancy.

The patient underwent surgery on the rotator cuffs and was then placed on a course of clinical and radiological surveillance every 6 months. There was nothing of note in the clinical check-up 6 months later and the patient did not complain of any residual pain. The scanner showed an indented humeral head with incisions in the front cortical bone at two points, without a “nidus”. At the 1-year check-up the MRI scan found a slightly raised area resembling a post-operative reformation. The decision not to operate on the osteoblastoma was maintained with image monitoring every 6 months, except in the case of an adverse clinical development.

Five years after this episode, the patient is still under surveillance. He does not present any functional problems or radiological changes of the existing lesion.

## Discussion

Our patient raises two issues: the rupture of the rotator cuff and the bone lesion.

The rupture of the rotator cuff does not cause any functional disability in our patient. The absence of correlation between the anatomical lesion and the significance of the symptoms is found in literature [1]. In our case, we cannot know whether the rupture of the rotator cuffs is a result or independent of the bone lesion. The fact that surgery was carried out without touching the bone suggests that it is not a result. Long-term monitoring without a reoccurrence of the tear reinforces this idea.

Osteoblastomas are an uncommon type of benign osteoid osteoma-like tumor and represent 1% of all bone tumors [2]. They develop from an excessive growth in the osteoid tissue with abundant vascular connective tissue and peripheral fibrous lesions [3]. This tumor affects men between 10 and 30 years of age, diagnosed before the age of 30 in over 90% of cases [4-6]. The literature describes osteoblastomas in a number of places, mainly on the axial skeleton (posterior vertebral arch in 40-50% of cases), and on some long bones (femur and tibia) but rarely on the humerus [4, 7]. Patient questioning and the clinical examination will typically identify strong pains at night, which do not react well to non-steroidal anti-inflammatory drugs but do react well to aspirin.

In our case, a 52-year-old man with a painless bone lesion, unusually on the humerus, raises the question of the diagnosis and that of the development of the lesion. Is this an osteoid osteoma or an osteoblastoma? Is there a risk that it will develop into a malignant tumor?

In the first instance, the diagnosis is based on the X-ray. In long bones, osteoblastomas are spindle-shaped osteolytic lesions that can break through the cortical bone with a moderate peripheral halo. Calcification can be found at the center of the osteolysis giving it a characteristic appearance [8]. In our case, the cortical bone did not break but thickened. The presence of a lesion of 3 mm in diameter supports the thesis of an osteoid osteoma. According to some authors, the distinction is found, among other things, in the “cutoff” size, where less than 15 mm would indicate an osteoid osteoma. Monitoring CT scan also supported the thesis of an osteoid osteoma given the absence of a “nidus” [9].

The positive diagnosis was obtained through a bone biopsy carried out under scan control. The anatomopathological analysis provided arguments in favor of an osteoblastoma due to the abundance of osteoblasts. With regard to osteoblastomas, current cytogenetic research has found specific markers that do not affect the form of treatment suggested and the prognosis [10].

Surgical treatment is necessary in the event of disabling pain or the aggressive growth of the tumor. It can be carried out by means of curettage or complete resection, with or without bone implants. Surgical resection seems to be more effective in avoiding relapse but certain studies suggest a risk of malignant transformation after curettage [11]. Treatments using radiofrequency seem to be very promising [12]. In our case, the absence of pain or functional disability supports the choice of keeping the patient under surveillance [13].

Osteoblastomas are benign tumors that in rare cases can develop into osteosarcoma- or osteochondroma-type tumors [6, 14]. This supports our decision to continue active monitoring.

### Conclusion

We have presented a rare case found in the course of a general medicine consultation. In cases of bone lesion or in age groups at risk, complementary tests are necessary. A biopsy is needed in order to obtain a definitive diagnosis. Even if clinical and X-ray tests suggest that the lesion is benign, we cannot always make the distinction between an osteoid osteoma and an osteoblastoma. This diagnosis is important in order to determine the course that should be followed. The risk of malignant transformation justifies active monitoring.
